# Down-regulation of *LAPTM5* in human cancer cells

**DOI:** 10.18632/oncotarget.8614

**Published:** 2016-04-06

**Authors:** Michelle Nuylan, Tatsuyuki Kawano, Johji Inazawa, Jun Inoue

**Affiliations:** ^1^ Department of Molecular Cytogenetics, Medical Research Institute and Graduate School of Medical and Dental Science, Tokyo Medical and Dental University, Tokyo, Japan; ^2^ Department of Esophageal and General Surgery, Graduate School, Tokyo Medical and Dental University, Tokyo, Japan; ^3^ Department of Genome Medicine, Hard Tissue Genome Research Center, Tokyo Medical and Dental University, Tokyo, Japan; ^4^ Bioresource Research Center, Tokyo Medical and Dental University, Tokyo, Japan

**Keywords:** LAPTM5, ESCC, tumor suppressor genes, lysosomes, cell death

## Abstract

Lysosomal-associated protein multispanning transmembrane 5 (*LAPTM5*) is a membrane protein that localizes to intracellular vesicles. It has been previously demonstrated that *LAPTM5* expression level is decreased in neuroblastoma (NB) cells, and excessive accumulation of LAPTM5 was shown to induce lysosomal cell death in these cells. However, the pathological expression and role of *LAPTM5* in other types of human cancers are largely unknown. Here, we found that *LAPTM5* mRNA level is frequently decreased in various cancer cell lines, and its low expression in patients with esophageal squamous cell carcinoma (ESCC) and non-small cell lung cancer (NSCLC) was significantly correlated with poor prognosis. Furthermore, we showed that overexpression of LAPTM5 in several cancer cells induces lysosomal cell death due to lysosomal destabilization, indicated by leakage of lysosomal cathepsin D into the cytosol as well as impairment of autophagy. These findings suggest that the inactivation of *LAPTM5* may contribute to tumorigenesis in a subset of human cancers.

## INTRODUCTION

Circumventing programmed cell death (PCD) is a common action of cancer cells; hence, tumor suppressor genes (TSGs) associated with PCD are frequently inactivated by genetic or epigenetic mechanisms in human cancer cells [1]. Characterizing the molecular mechanism of TSG-mediated PCD is crucial not only for understanding the pathological process of tumorigenesis, but also for the development of novel therapeutic approaches.

The *LAPTM5* (Lysosomal-associated protein multispanning transmembrane 5) gene encodes a membrane protein that localizes to intracellular vesicles, regulating vesicle trafficking such as endocytosis, and is highly expressed in lymphoid lineage cells [2–4]. Furthermore, LAPTM5 can inhibit the expression of T cell receptor (TCR), B cell receptor (BCR), and pre-B cell receptor (pre-BCR) on the cell surface *via* promotion of lysosomal degradation of these proteins; LAPTM5 deficiency has been shown to activate T cells or B cells due to an increase in level of their respective receptors [4, 23, 24]. Thus, previous reports have suggested that LAPTM5 may play an important role as a negative regulator of T cell or B cell receptor-mediated signaling [4, 23, 24].

We previously reported that this gene was transcriptionally down-regulated in neuroblastoma (NB) cell lines and primary tumors by DNA methylation around its transcriptional start site [5]. Interestingly, while LAPTM5 is continuously transported to lysosomes for degradation, it is accumulated in NB cells undergoing PCD within tumors with a favorable prognosis, which frequently undergo spontaneous regression. Furthermore, we have demonstrated that overexpression of LAPTM5 in NB cells induces lysosomal cell death due to lysosomal destabilization, indicated by leakage of lysosomal cathepsin D into the cytosol via lysosomal membrane permeabilization (LMP), as well as by impairment of autophagy degradation [5]. The E3 ubiquitin ligase *ITCH*, which is often amplified in chromosome 20q of undifferentiated thyroid tumors, is an oncogene [6] and can suppress the expression level of LAPTM5 via ubiquitination-dependent proteasomal degradation [7]. Expression of ITCH also abrogates LAPTM5-induced cell death in NB cells [7]. Thus, it has been suggested that *LAPTM5* expression and accumulation may contribute to PCD during NB tumor regression, and that *LAPTM5* may function as a tumor suppressor in NB cells. However, the pathological expression and role of LAPTM5 in other types of human cancers remain largely unknown.

Here, we report that expression of *LAPTM5* is frequently decreased at the transcriptional level in various types of human cancer cell lines and in non-small cell lung cancer (NSCLC) and esophageal squamous cell carcinoma (ESCC) tumors, and low expression is associated with poor prognosis of patients with such tumors. Importantly, we showed that overexpression of LAPTM5 induces lysosomal cell death in KYSE170 cells, an ESCC cell line, as well as in NB cells [5]. These findings suggest that inactivation of *LAPTM5* may contribute to tumorigenesis in a subset of human cancers.

## RESULTS

### Down-regulation of *LAPTM5* expression in human cancer cells

**Figure 1:**
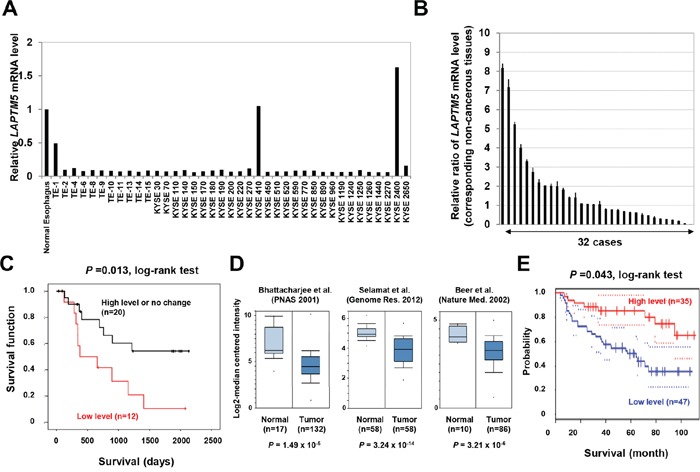
Down-regulation of *LAPTM5* in human cancers **A** and **B.** Expression analysis of *LAPTM5* in ESCC cell lines and primary samples. The mRNA level of *LAPTM5* in normal esophageal tissue, in 39 ESCC cell lines, and in 32 ESCC paired samples of primary tumors and corresponding non-cancerous tissues were measured by qRT-PCR. Expression of *GAPDH* was used as an internal control. The graph shows the relative expression of *LAPTM5* in the ESCC cell lines (relative to that in the normal esophageal tissue) (**A**) or in the primary tumor (relative to that in the corresponding non-cancerous tissue) (**B**). Bar; standard deviation (SD). **C.** Kaplan–Meier curves for overall survival rates of the 32 patients with ESCC, according to *LAPTM5* expression status (high or low expression). Low expression (more than 30% reduction of *LAPTM5* expression) was significantly associated with worse survival by log-rank test. **D.** Expression levels of *LAPTM5* in lung adenocarcinoma. Three lung adenocarcinoma gene expression datasets were analyzed in Oncomine and displayed as a box plot (log_2_ median-centered). *P* values were calculated using Student's *t*-test. **E.** Correlation between *LAPTM5* expression and prognosis among lung cancer patients. Correlation between *LAPTM5* expression and survival was analyzed and plotted using the Kaplan–Meier method. Survival rates for patients with high (n=35) and low (n=47) *LAPTM5* expression were analyzed using PrognoScan. *P* values were calculated by log-rank test. The 95% confidence intervals for each group are indicated by dotted lines.

We first examined the expression status of *LAPTM5* in 333 cell lines from 18 various types of human cancers in comparison with the expression levels of each corresponding normal tissue by qRT-PCR analysis. As shown in Table 1, Figure 1A, and Figure S1, we found that the expression level of *LAPTM5* mRNA was decreased in 316 of the 333 cell lines (94.9%), including in 37 of the 39 ESCC cell lines (94.9%). A qRT-PCR analysis of paired ESCC samples (primary tumors and their corresponding non-cancerous tissues) revealed a more than 30% reduction of *LAPTM5* expression in primary tumor tissues compared with the corresponding non-cancerous tissues in 12 of the 32 cases (37.5%) (Figure 1B). Importantly, the Kaplan-Meier survival curve indicated that low expression of *LAPTM5* was significantly associated with poor prognosis of patients with ESCC (*P* = 0.013) (Figure 1C). However, apart from poor prognosis, we could not show any correlation between low expression and other clinical implications such as stages, pathological grades, or lymph metastasis (Table S1). In addition, we could not detect aberrant DNA methylation around the transcriptional start site of *LAPTM5* in the ESCC tumor samples showing low expression, as is detected in NBs (data not shown) [5]. Furthermore, we surveyed 3 gene-expression datasets of NSCLC adenocarcinomas using Oncomine, a cancer microarray database (www.oncomine.org/). *LAPTM5* expression was decreased in NSCLC compared with normal lung tissues, and this difference was statistically significant in all 3 datasets (*P* = 1.49×10^−5^, *P* = 3.24×10^−14^, and *P* = 3.21×10^−6^) (Figure 1D) [13–15]. Additionally, we also showed that low expression of *LAPTM5* was significantly correlated with poor prognosis in NSCLC patients by analysis of lung cancer gene expression datasets that included survival information (*P* = 0.043) (Figure 1E) [16]. These findings indicate that *LAPTM5* is widely down-regulated in most human cancers, and notably, its down-regulation may serve as a poor prognostic factor for patients with ESCC and NSCLC.

**Table 1:** Frequency of down-regulation of LAPTM5 expression in human cancer cell lines

Tissue	Number of down-regulated cell lines *	Number of total cell lines	Frequency (%)
Esophageal Squamous Cell Carcinoma (ESCC)	37	39	94.9
Prostate	3	3	100
Anaplastic Thyroid Carcinoma (ATC)	15	16	93.8
Cervical	11	11	100
Hepatic Cellular Carcinoma (HCC)	21	25	84
Breast	13	13	100
Glioma	21	23	91.3
Renal Cell Carcinoma (RCC)	13	15	86.7
Endometrial	14	14	100
Ovarian	22	27	81.5
Bladder	8	8	100
Gallbladder	8	8	100
Small Cell Lung Carcinoma (SCLC)	21	21	100
Gastric	29	30	96.7
Mesothelial	4	4	100
Myeloma	10	10	100
Non-Small Cell Lung Carcinoma (NSCLC)	35	35	100
Pancreatic	31	31	100
Total	316	333	94.9

“Down-regulation” was defined as a 50% or greater reduction in expression compared with the corresponding normal tissue.

### Induction of cell death by overexpression of LAPTM5 in cancer cells

**Figure 2:**
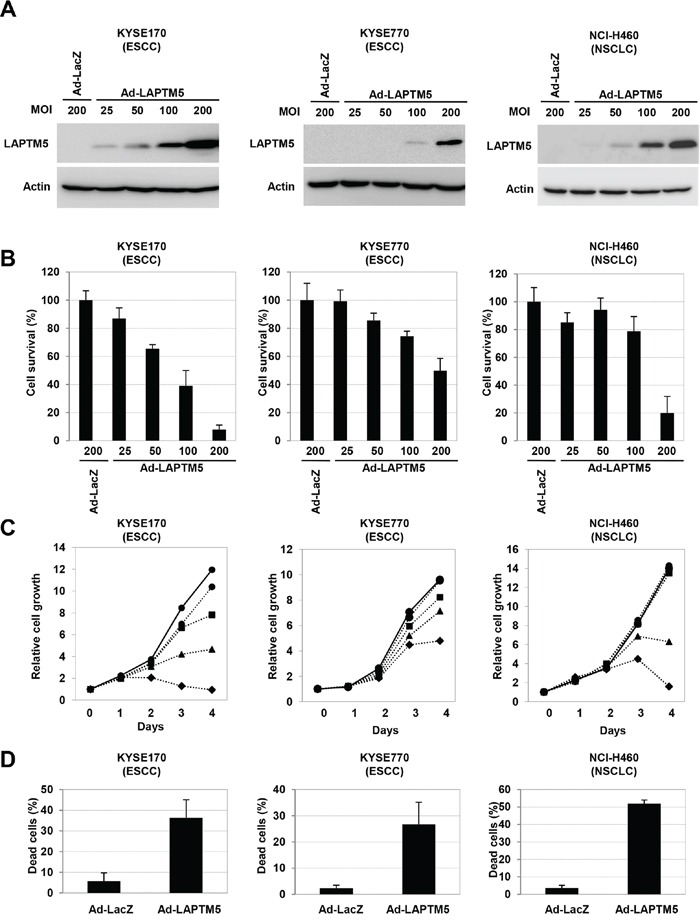
Overexpression of LAPTM5 induced cell death in cancer cell lines **A.** Western blot analysis of LAPTM5-expressing cells. Cells were infected with Ad-LacZ or Ad-LAPTM5 for 4 days at the indicated MOI. Whole cell lysates were subjected to SDS-PAGE and immunoblotted with rabbit anti-LAPTM5 or mouse anti-β-actin antibody. **B.** Effect of LAPTM5 overexpression on cell survival in cancer cells. Cells were infected with Ad-LacZ or Ad-LAPTM5 for 4 days at the indicated MOI. The cell survival rate relative to that of Ad-LacZ-infected cells was assessed by the CV staining assay. Bar; standard deviation (SD) for triplicate experiments. **C.** Cell growth assay. Growth curves are indicated by solid lines for Ad-LacZ-infected cells or dotted lines for Ad-LAPTM5-infected cells. Ad-LacZ was infected at 200 MOI. Ad-LAPTM5 was infected at 25 (circle), 50 (square), 100 (triangular), or 200 (quarry) MOI. **D.** Frequency of dead cells. KYSE170 cells were infected with Ad-LacZ or Ad-LAPTM5 at 200 MOI. Four days after infection, dead cells were counted using the trypan blue exclusion method, and indicated as percentages. Bar; standard deviation (SD) for triplicate experiments.

To test whether overexpression of LAPTM5 can induce cell death in other types of cancer cell lines, as in NB cells [5], LAPTM5 was introduced by adenovirus into KYSE170 and KYSE770 cells (both ESCC cell lines) and NCI-H460 cells (a NSCLC cell line), all of which demonstrate down-regulation of *LAPTM5* (Figure 1A and Figure S1). Western blot analysis revealed that LAPTM5 expression gradually increased with increasing adenovirus-LAPTM5 (Ad-LAPTM5) titer (Figure 2A). Overexpression of LAPTM5 for 4 days resulted in remarkable decreases in cell survival in an Ad-LAPTM5 dose-dependent manner (Figure 2B). In addition, more severe growth inhibition and a remarkable increase of dead cells were observed in cells infected with higher Ad-LAPTM5 titer, compared with adenovirus-LacZ (Ad-LacZ) (Figure 2C and 2D). Consistent with NB cells [5], there were no typical features of apoptosis, such as caspase activation or apoptotic bodies, in ESCC and NSCLC cells undergoing LAPTM5-induced cell death (data not shown). These results suggest that overexpression of LAPTM5 can effectively induce non-apoptotic cell death in human cancer cells.

### Induction of lysosomal destabilization during LAPTM5-mediated cell death

**Figure 3:**
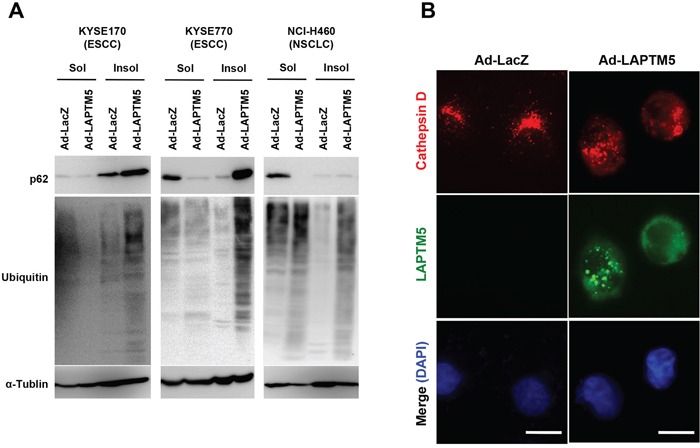
Induction of lysosomal destabilization during LAPTM5-mediated cell death **A.** Western blotting for ubiquitinated proteins. Four days after infection in KYSE170, KYSE770, or NCI-H460 cells, cells were lysed in 0.1% Triton-X solution. Triton-insoluble pellets were solubilized in 2% SDS buffer. Triton-X soluble or -insoluble lysates were loaded onto a 6% SDS-PAGE and analyzed by immunoblotting with antibodies against p62, ubiquitin, and α-tubulin. In both soluble and insoluble samples, greater accumulation of p62 and ubiquitinated proteins were observed in Ad-LAPTM5-infected cells compared with Ad-LacZ-infected cells. **B.** Representative images of cathepsin D staining. Four days after infection, KYSE170 cells were fixed in 10% TCA, reacted with anti-cathepsin D and anti-LAPTM5 antibodies, and visualized with Alexa Fluor 594-conjugated mouse IgG and Alexa Fluor 488-conjugated rabbit IgG antibodies, respectively. Bar, 10 μm.

We next examined whether lysosomal destabilization is induced by overexpression of LAPTM5 in other types of cancer cell lines, as is in NB cells [5]. Because p62/SQSTM1 (referred to as p62) and ubiquitinated proteins are continuously degraded in the lysosome via an autophagic process, lysosomal dysfunction or autophagy inhibition leads to an accumulation of these proteins in the Triton-X-insoluble fraction [17–22]. As shown in Figure 3A, levels of p62 and ubiquitinated proteins were clearly increased in the Triton-X-insoluble fraction from LAPTM5-expressing cells, compared with that of the Triton-X-soluble fraction. Additionally, we showed that overexpression of LAPTM5 induced leakage of lysosomal cathepsin D into the cytoplasm in KYSE170 cells, indicated by a change in cathepsin D localization from a punctate to a diffusive pattern, suggesting lysosomal membrane permeabilization (LMP), an indicator of lysosomal destabilization (Figure 3B). Taken together, these findings suggest that overexpression of LAPTM5 protein induces lysosomal destabilization, accompanied by autophagy impairment and cathepsin D leakage, resulting in the lysosomal cell death of ESCC and NSCLC cells.

## DISCUSSION

We showed that overexpression of LAPTM5 induces lysosomal cell death, demonstrated by cathepsin D release from the lysosome into the cytoplasm, as well as impairment of autophagy via lysosomal destabilization in ESCC and NSCLC cells, as was previously observed in NB cells [5]. This suggests that LAPTM5-induced cell death may widely occur in various human cancers. However, the detailed molecular mechanism underlying LAPTM5-induced cell death remains unknown. We previously demonstrated that LAPTM5 protein levels were negatively regulated not only by lysosomal degradation, but also by proteasomal degradation via ITCH, an E3 ubiquitin ligase, in NB cells [5, 7]. We demonstrated that accumulation of LAPTM5 due to dysfunction of these degradation pathways was critical for the induction of cell death in NB cells [5, 7]. We also showed that LAPTM5 was accumulated in dying NB cells within tumors undergoing spontaneous regression [5]. In the present study, we showed that overexpression of LAPTM5 protein by a high-dose of adenovirus could induce cell death in ESCC and NSCLC cells. Thus, these findings suggest that excessive accumulation of LAPTM5 may be a triggering event for induction of cell death in human cancers.

Moreover, we observed that *LAPTM5* expression was frequently decreased at the transcriptional level in various types of human cancer cells, similar to NB cells [5]. In particular, *LAPTM5* down-regulation was significantly correlated with poor prognosis of patients with ESCC and NSCLC. We have previously reported that aberrant DNA methylation around the transcriptional start site (TSS) of *LAPTM5* may be a possible mechanism for down-regulation of *LAPTM5* in NB cells [5]. However, we were unable to detect aberrant methylation in this region in ESCC tumors that showed low expression of *LAPTM5* (data not shown), suggesting that other mechanisms, such as chromatin modification around the TSS, chromosomal deletion, or loss of transcriptional factors, may be closely associated with the down-regulation of *LAPTM5* expression in ESCC tumors. Thus, further validations including somatic mutation will be required for understanding molecular mechanism for inactivation of *LAPTM5* gene in human cancers.

Finally, our findings suggest that the transcriptional down-regulation of *LAPTM5* may lead to the repression of LAPTM5 accumulation-mediated cell death in human cancers, including ESCC and NSCLC. Although we have observed accumulation of LAPTM5 in dying cells within regressing NB tumors, it is unknown whether this accumulation actually occurs in other tumors, including ESCC. Thus, identifying the physiological and cellular conditions of LAPTM5 accumulation-mediated cell death will be essential to further understand the biological significance of this type of cell death in human cancers.

## MATERIALS AND METHODS

### Cell culture and primary tumor samples

Cell lines were obtained from the American Type Culture Collection (ATCC) or from the Japanese Collection of Research Bioresources (JCRB). ESCC cell lines, which were given by Dr. Yataka Shimada of Kyoto University [8–10], and NCI-H460 (an NSCLC cell line) were cultured in RPMI1640 medium containing 10% FBS. All cell lines were maintained at 37°C with 5% CO_2_.

A total of 32 primary ESCC tumor samples and their corresponding non-cancerous esophageal mucosa were investigated in this study. All samples were obtained from patients treated at the Tokyo Medical and Dental University Hospital between November 2007 and October 2012, were frozen immediately in liquid nitrogen, and stored at −80°C until total RNA was extracted. The collection and analysis of patient samples were approved by the Tokyo Medical and Dental University Institutional Review Board (approval #2010-5-4), and written consent was obtained from all patients.

### Antibodies

Antibodies against β-actin (A5441, Sigma), α-tubulin (Sigma), p62 (SQSTM1; sc-28359, Santa Cruz Biotechnology), Ubiquitin (Wako), Cathepsin D (IM03, Calbiochem), and LAPTM5 (H-178, Santa Cruz Biotechnology) were used for western blotting and immunofluorescence analysis.

### Recombinant adenovirus

Replication-defective recombinant adenoviruses were constructed with the Adenovirus Expression Vector Kit (Takara) following the manufacturer's recommendations [5]. Viral titers were measured in pfu/ml by a serial dilution method using HEK293 cells. Cells were infected with each indicated MOI (multiplicity of infection; PFU/cell).

### RNA isolation and quantitative reverse transcription (qRT)-PCR

RNA isolation and qRT-PCR were performed as described in previous reports [11] for the expression analysis of 338 cell lines and the corresponding normal tissues, as shown in Supplementary Figure 1. Total RNA was isolated using TRIzol^®^ reagent (Invitrogen) according to standard procedures. Single-stranded cDNA generated from total RNA was amplified with primer sets specific for each gene. For quantitative real-time RT–PCR, probes for *LAPTM5* were purchased from the KAPA PROBE FAST system (KAPA BIOSYSTEMS). RT–PCR was performed using an ABI PRISM 7500 sequence detection system (Applied Biosystems) according to the manufacturer's instructions. Gene expression values are given as ratios (differences between the Ct values) between the gene of interest and an internal reference (*GAPDH*) that provides a normalization factor for the amount of RNA isolated from a specimen, which was then normalized by the control cell value (relative expression level).

### Western blotting analysis

Western blotting was performed as described in previous reports [5, 12]. Whole cell lysates were subjected to SDS-PAGE, and proteins were transferred to PVDF membranes (GE Healthcare). After blocking with TBS containing 0.05% Tween-20 and 5% non-fat dry milk for 1 hour, the membrane was reacted with an antibody overnight. The dilutions for primary antibodies were: 1/5,000 for β-actin, 1/2,000 for p62, and 1/1,000 for LAPTM5 and Ubiquitin. The membrane was washed and exposed to horseradish peroxidase (HRP)-conjugated anti-mouse or rabbit IgG antibodies (both at 1/4,000) for 2 hours. The bound antibodies were visualized in LAS3000 (GE Healthcare) using a Pierce ECL Western detection kit according to the manufacturer's instructions (Thermo Scientific). Alternatively, cells were lysed in 0.1% Triton-X solution and the Triton-X-insoluble pellet was solubilized in 2% SDS buffer. Triton-X soluble or -insoluble lysates were loaded onto a 6% SDS-PAGE and analyzed by immunoblotting with p62, ubiquitin, or α-tubulin antibodies.

### Cell survival and cell death assays

Cell survival and cell death assays were performed as previously described [5, 12]. Cell survival was assessed by the crystal violet (CV) staining assay. Cells were washed in PBS and fixed with 0.1% CV in 10% formaldehyde in PBS for 10 minutes. After excess CV solution was discarded, stained cells were completely air-dried, and then lysed with a 2% SDS solution with shaking for 2 hours. Optical density (OD) absorbance was measured at 560 nm using a microplate reader (ARVOmx; PerkinElmer), and the percent absorbance of every well was determined. The OD absorbance of cells in control wells was arbitrarily set at 100% to determine percentages of viable cells. Dead cells were counted by trypan blue staining using the TC20™ Automated Cell Counter (BIO-RAD), and the percent dead cells of total cells was calculated.

### Immunofluorescence (IF) analysis

IF analysis was performed as described from previous reports [5, 7]. Cells were fixed in 10% trichloroacetic acid and permeabilized with 0.5% Triton X-100 in PBS. After blocking with PBS containing 1% bovine serum albumin and 0.01% Triton X-100 for 1 h at 4°C, the cells were incubated with mouse anti-Cathepsin D (dilution: 1/1000) overnight at 4°C. Bound antibody was visualized using Alexa Fluor 488 anti-mouse IgG antibody (Invitrogen, dilution: 1/2000). The cells were mounted in VECTASHIELD Mounting Medium with DAPI (Vector Laboratories) and observed under a fluorescence microscope (DM6000 B, Leica, Wetzlar, Germany).

### Analysis of gene expression datasets

Three gene expression datasets of human lung cancers [13–15] were analyzed in Oncomine, a cancer microarray database (www.oncomine.org/). Kaplan–Meier analysis of 82 lung cancer patients was based on the PrognoScan database (http://www.prognoscan.org/) using the publicly available Gene Expression Omnibus (http://www.ncbi.nlm.nih.gov/geo), with the accession number GSE68465 [16]. *P* values were calculated by log-rank test.

### Statistical analysis

Differences between subgroups were tested by Student's t-test. A *P* value of < 0.05 was considered statistically significant.

## SUPPLEMENTARY FIGURE AND TABLE




