# Supplementary material for: Down-regulation of LAPTM5 in human cancer cells
Source: Oncotarget. 2016 Apr 6;7(19):28320–8. doi: 10.18632/oncotarget.8614 (PMC5053729; doi:10.18632/oncotarget.8614)
Supplement: Supplementary file 1 [file oncotarget-07-28320-s001.pdf]

Down-regulation of *LAPTM5* in human cancer cells

SUPPLEMENTARY FIGURE AND TABLE

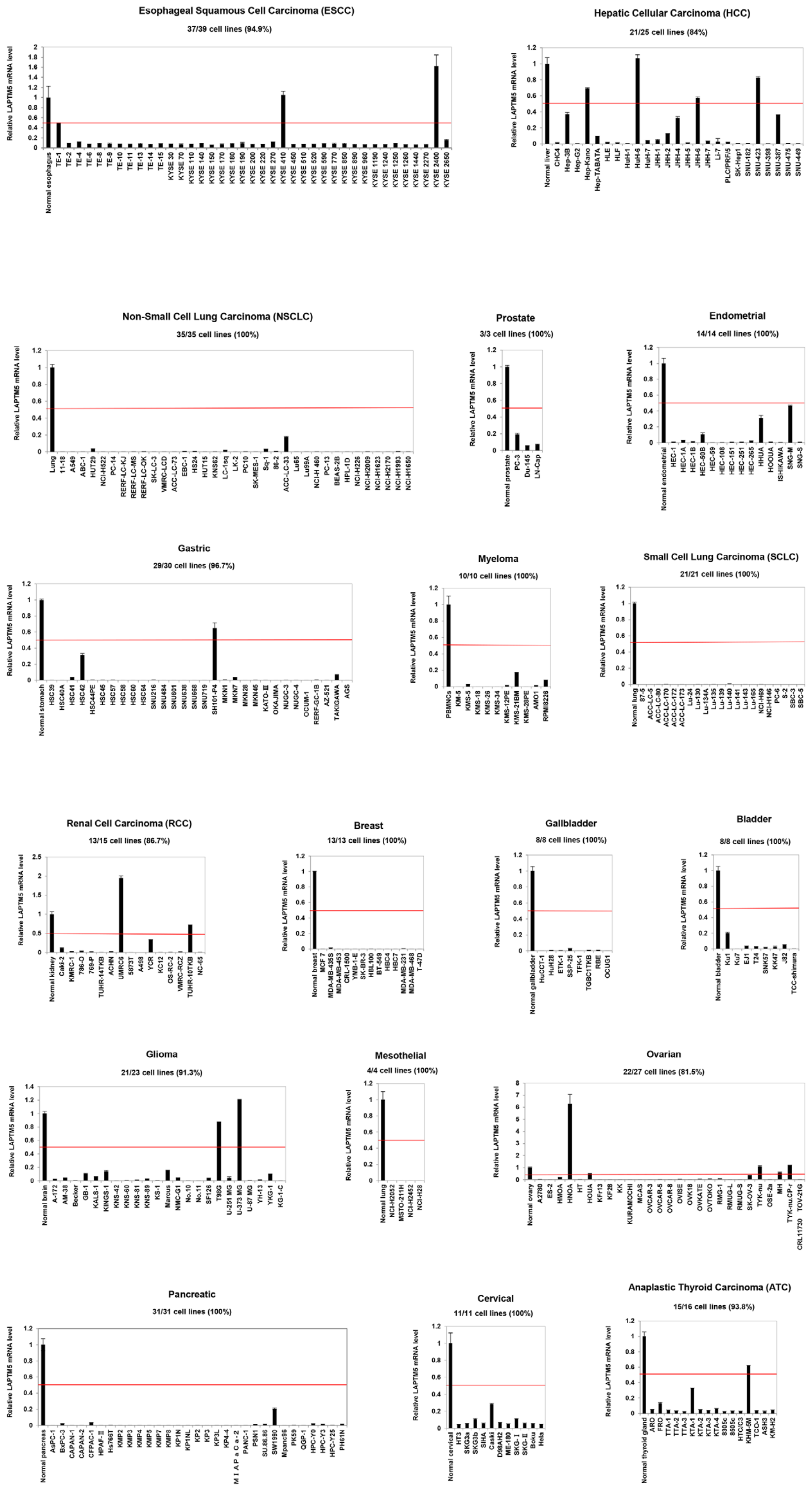

Supplementary Figure S1: Expression analysis of *LAPTM5* in human cancer cell lines. The mRNA levels of *LAPTM5* in 333 cancer cell lines and their corresponding normal tissues were measured by qRT-PCR. Expression of *GAPDH* was used as an internal control. Each graph indicates the relative expression of *LAPTM5*, compared with the normal tissue value arbitrarily set to 1.0. Bar; standard deviation (SD). Down-regulation was defined as a 50% or greater reduction in expression.

Supplementary Table S1: Correlation between clinicopathological background and down-regulation of LAPTM5 expression

See Supplementary File 1
